# Evaluating background and local contributions and identifying traffic-related pollutant hotspots: insights from Google Air View mobile monitoring in Dublin, Ireland

**DOI:** 10.1007/s11356-024-34903-5

**Published:** 2024-09-10

**Authors:** Jiayao Chen, Anna Mölter, José Pablo Gómez-Barrón, David O’Connor, Francesco Pilla

**Affiliations:** 1 School of Architecture, Planning and Environmental Policy, University College Dublin, Dublin, Ireland; 2 School of Chemical Sciences, Dublin City University, Dublin, Ireland; 3 School of Biosystems and Food Engineering, University College Dublin, Dublin, Ireland

**Keywords:** Mobile monitoring, Traffic-related pollutants, Spatiotemporal variation, Background contribution, Short- and long-lived events, Hotspot analysis

## Abstract

Mobile monitoring provides high-resolution observation on temporal and spatial scales compared to traditional fixed-site measurement. This study demonstrates the use of high spatio-temporal resolution of air pollution data collected by Google Air View vehicles to identify hotspots and assess compliance with WHO Air Quality Guidelines (AQGs) in Dublin City. The mobile monitoring was conducted during weekdays, typically from 7:00 to 19:00, between 6 May 2021 and 6 May 2022. One-second data were aggregated to 377,113 8 s road segments, and 8 s rolling medians were aggregated to hourly and daily levels for further analysis. We assessed the temporal variability of fine particulate matter (PM_2.5_), nitrogen monoxide (NO), nitrogen dioxide (NO_2_), ozone (O_3_), carbon monoxide (CO), and carbon dioxide (CO_2_) concentrations at hyperlocal levels. The average daytime median concentrations of NO_2_ (28.4 ± 15.7 µg/m^3^) and PM_2.5_ (7.6 ± 4.7 µg/m^3^) exceeded the WHO twenty-four hours (24 h) Air Quality Guidelines in 49.4% and 9% of the 1-year sampling time, respectively. For the diurnal variation of measured pollutants, the morning (8:00) and early evening (18:00) showed higher concentrations for NO_2_ and PM_2.5_, mostly happening in the winter season, while the afternoon is the least polluted time except for O_3_. The low-percentile approach along with 1-h and daytime minima method allowed for decomposing pollutant time series into the background and local contributions. Background contributions for NO_2_ and PM_2.5_ changed along with the seasonal variation. Local contributions for PM_2.5_ changed slightly; however, NO_2_ showed significant diurnal and seasonal variability related to traffic emissions. Short-lived event enhancement (1 min to 1 h) accounts for 36.0–40.6% and 20.8–42.2% of the total concentration for NO_2_ and PM_2.5_. The highly polluted days account for 56.3% of total NO_2_, highlighting local traffic is the dominant contributor to short-term NO_2_ concentrations. The longer-lived events (> 8 h) enhancement accounts for 25% of the monitored concentrations. Additionally, conducting optimal hotspot analysis enables mapping the spatial distribution of “hot” spots for PM_2.5_ and NO_2_ on highly polluted days. Overall, this investigation suggests both background and local emissions contribute to PM_2.5_ and NO_2_ pollution in urban areas and emphasize the urgent need for mitigating NO_2_ from traffic pollution in Dublin.

**Supplementary Information:**

The online version contains supplementary material available at 10.1007/s11356-024-34903-5.

## Introduction

Air pollution is recognized as a public health emergency for humans. Multiple epidemiological studies have been conducted globally, with evidence showing adverse health effects and premature death linked to short-term and long-term exposure to particulate matter and gas pollutants (Chen and Kan 2008, Nuvolone et al. 2018; Samoli et al. 2001, World Health Organization 2004, 2013, 2016). Consistent research findings were shown from studies that were conducted in Ireland. Since the implementation of the Air Pollution Act in the 1990s, Ireland has experienced a decline in ambient concentrations of particulate matter, nitrogen dioxide (NO_2_), and carbon monoxide (CO) (Carthy et al. 2020; Clancy et al. 2002), and the health benefits of air pollution controls showing reduced respiratory and cardiovascular deaths in Dublin. However, more recent research evidence highlighted there was no safe level of air pollutants to cause adverse effects. Quintyne et al. (2020) found the linkage of poor air quality with a rise in hospital admissions for asthma, chronic obstructive airway disease, and heart failure in Dublin city. Specifically, associations of outdoor particulate matter exposure with premature death from type 2 diabetes and cardio-respiratory diseases have been reported (McVicar et al. 2023; White et al. 2020). For example, Carthy et al. (2020) highlighted the associations between NO_2_ pollution and asthma in older adults in Ireland. All stoke admission risks in Dublin were associated with increased PM_2.5_ and NO_2_ pollution during the winter season (Byrne et al. 2020), suggesting the exceedance of these pollutants in the short term could lead to acute health problems. The persistent efforts to mitigate air pollution, even at relatively lower levels, will continue to yield health benefits for the public.

In September 2021, the World Health Organization (WHO) launched stringent Air Quality Guidelines (AQGs) for major air pollutants, including inhalable particulate matter (PM_10_, with particle size equal to or less than 10 μm), fine particulate matter (PM_2.5_, with particle size equal to or less than 2.5 μm), NO_2_, ozone (O_3_), sulfur dioxide, and CO to provide better protection for the general public (WHO 2021). Nearly, the whole global population (99%) breathes fine particles (PM_2.5_) that exceed the new WHO AQGs annual level (5 µg/m^3^) (Yu et al. 2023). European Environment Agency (2022) estimated that 96% of the urban population in Europe was exposed to PM_2.5_ above the AQGs in 2020. As part of the European Union (EU) Green Deal, the EU Commission proposed to revise the Directive on Ambient Air Quality to align with the recently updated WHO AQGs (Tankosić 2023). To adhere to the legislation and EU directives, it is critical to maintain engagement in evidence-based policymaking, effectively reducing air pollution for the benefit of public health in Ireland.

Air quality characterization at a global or regional scale was mostly assessed using remote sensing (Brown et al. 2021). These methods have also been employed at the national or local level, for example, Kumari et al. (2022) employed satellites and ground-based data to characterize the air quality during the COVID lockdown in Dublin. Additionally, statistical models (e.g., linear or non-linear) using a small number of fixed site measurements in combination with geographic and temporal parameters have been developed to predict air pollutants at the city level (Alam and McNabola 2015, Basu et al. 2019; Donnelly et al. 2019). These studies are limited by a need for high-resolution data. Notably, most epidemiological studies regarding air pollution and health effects are generally based on ambient concentrations measured from limited representative regulatory stations or derived from modeling results (McDuffie et al. 2021). In complement to conventional methods, mobile monitoring proves to be an effective approach to assessing the temporal and spatial variabilities of air pollutants at a fine scale.

Google Street View vehicles equipped with air sensors have been employed for comprehensive traffic air pollution investigation in North America (e.g., Denver, San Diego, Oakland) and European cities (e.g., Amsterdam, Copenhagen, London) (Alexeeff et al. 2018; Apte et al. 2017; Kerckhoffs et al. 2022; Whitehill et al. 2020). Along with the rapid economic growth, urbanization, and increasing number of private cars (Tubridy et al. 2022), people in Dublin experience bad traffic congestion similar to that of other European citizens. Road traffic emission represents a major source of PM_2.5_, CO, and NO_2_, with nitrogen oxides (NOx) serving as one of the main precursors of O_3_ formation. Severe traffic congestion could lead to toxic levels of traffic-related air pollutants (Jin and Jin 2023), and there was evidence indicating that health damage could be attributed to diesel vehicles in Dublin, Ireland (Dey et al. 2018). Ireland’s national and local air quality monitoring networks are essential to ensure compliance with air quality standards. However, limited studies provided a microscale level assessment of traffic-related air pollutants. Responding to this data gap, high-resolution monitoring of gas (CO, NO_2_, O_3_, nitrogen monoxide (NO), carbon dioxide (CO_2_)) and particulate pollutants (e.g., PM_2.5_) was performed using Google’s electric Street View car at street level in Dublin city, Ireland.

Using high-resolution criteria pollutants mapping allows a comprehensive understanding of the spatiotemporal variation of air pollution within Dublin city, thereby enabling the formulation of mitigation policy targeting localized air pollution phenomena. The objectives of this paper are to (1) assess the daytime trend, weekday variations, and seasonal patterns of criteria air pollutants; (2) assess the temporal variations of local and background contribution to time serial air pollution; and (3) address the spatial complexities of hyperlocal air quality. This meta-data enables real-time information about the exposure level and hotspots for further air quality management and public awareness. Furthermore, it is critical for a growing urgency for mitigation strategies and future environmental policy to meet the zero-pollution action plan for 2030 and the zero-pollution version for 2050.

## Methodology

### Study area

Dublin is the capital of the Republic of Ireland, with a population of 1.45 million in 2022 (Central Statistics Office 2022) and an approximate area of 118 km^2^. The Dublin city center is characterized by a mix of residential and commercial buildings. A study indicated that about 10.5% and 9.8% of Ireland’s national CO_2_ and PM_2.5_ emissions from traffic sources occurred in Dublin city (Alam et al. 2018). It has a transport network with national roads (N) and regional roads (R), tram (i.e., LUAS), train (i.e., DART), and cycle lanes. Dublin has a maritime climate without significant temperature extremes and is characterized by mild-warm summer (12–16 °C) and cool winter (4–8 °C) (McHugh et al. 2023). The prevailing wind direction in Ireland is from the southwest (Broderick et al. 2006).

### Measurement protocol and mobile air sensing platform

**Fig. 1:**
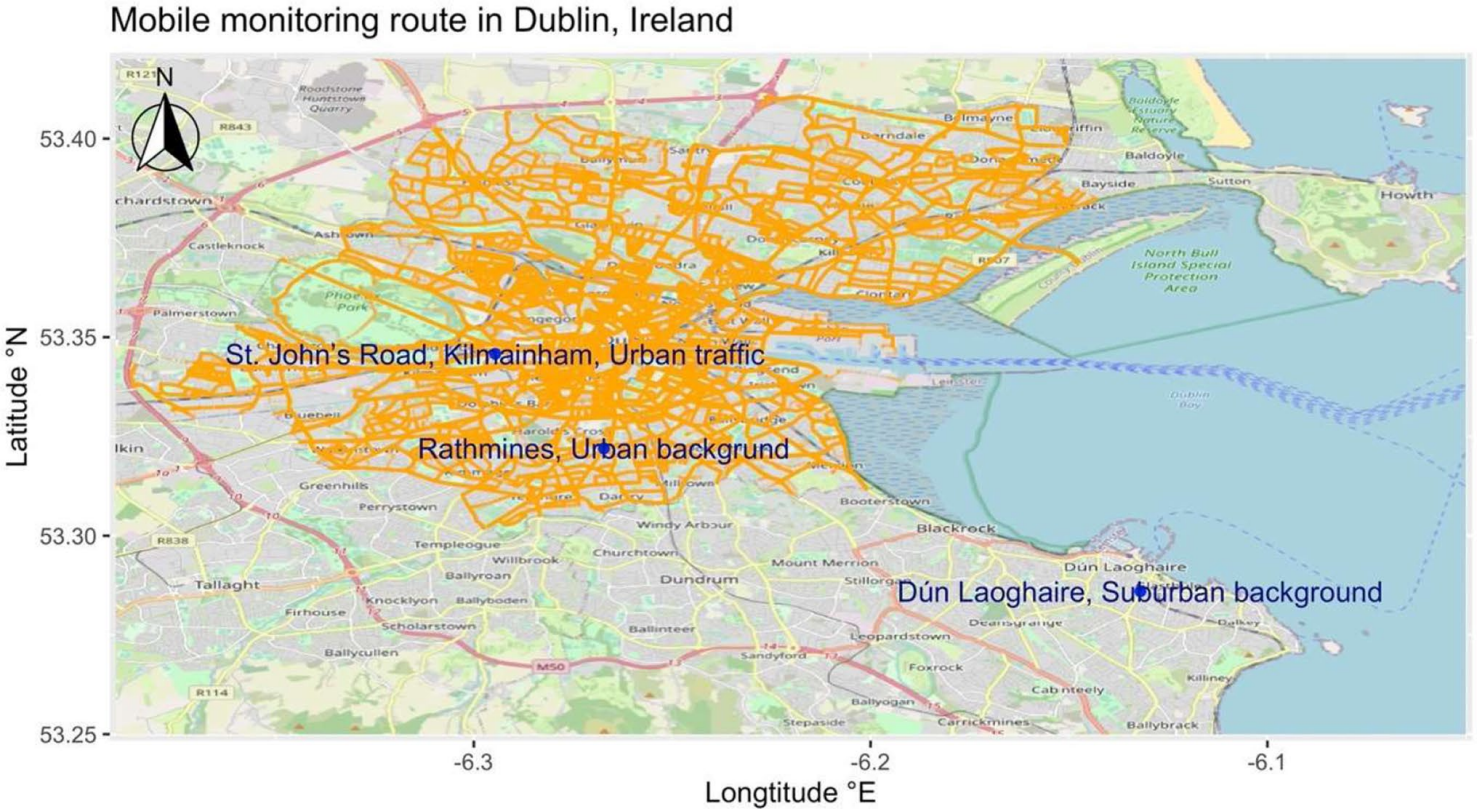
Overview of the “Google Air View” mobile monitoring routes (orange dots) in Dublin city from May 2021 to May 2022. The blue dots indicated the location of the urban background, suburban background, and urban traffic sites

A hyperlocal air pollutants monitoring campaign using an all-electric Street View vehicle (Jaguar I-PACE) equipped with Aclima’s mobile air measurement and data acquisition platform was set up in Dublin, Ireland, over 16 months. This Google Street View car was characterized by zero tailpipe emission. The Aclima’s mobile air sensing platform was driven through roads and streets in Dublin, following fixed routes and time slots to capture air pollutants at a high spatiotemporal resolution. In general, mobile monitoring was repeatedly conducted in Dublin city on weekdays from Monday to Friday during daytime hours, between 7:00 and 17:00, with a limited number of monitoring sessions extending until 18:00 and 19:00. A Global Positioning System (GPS) was used to record the geographical location, which was synthesized with the air monitoring platform for date and time. Figure 1 shows the mobile monitoring routes.

Aclima’s mobile measurement platform is equipped with laboratory-grade instruments, which are characterized by fast time-response (up to 1 Hz) air monitors that measure size-resolved particle number (PN) counts and NO_2_, NO, CO, and CO_2_, except O_3_ (3 s). PM_2.5_ concentrations were measured using the light scattering optical particle counter (Whitehill et al. 2020), which was reported along with PN in five size ranges of 0.3–0.5 µm, 0.5–0.7 µm, 0.7–1.0 µm, 1.0–1.5 µm, and 1.5–2.5 µm. Additionally, the real-time equipment measures NO (chemiluminescence), NO_2_ (cavity-attenuated phase shift spectroscopy), O_3_ (UV absorption), CO_2_ (non-dispersive infrared absorption), and CO (UV fluorescence).

This study focused on mobile monitoring data collected from May 2021 to May 2022. The metadata platform provides information about data collection, data management, data processing methods, measurement calibration, quality control and quality assurance (QA/QC) protocol, and uncertainty quantification. QA/QC procedures for the Google Air View project in Dublin and data processing protocols/algorithms in this study followed the reports of Apte et al. (2017) and Whitehill et al. (2020). Instrument performance including precision, bias, and drift for the monitored pollutants (NO, NO_2_, O_3_, CO, CO_2_, PM_2.5_) is shown in Table S1.

### Ambient monitoring data

Hourly PM_2.5_, NO_2_, and O_3_ concentrations measured from urban traffic, urban background, and suburban across Dublin were obtained from the national and local air quality networks (https://airquality.ie/). The ambient air quality data collected during the mobile monitoring period were incorporated for comparison. Information about the ambient sites can be referred to Table S2 of the Supporting Information (SI).

### Data cleaning and data aggregation

We performed a series of data processing and data aggregation steps to convert data into different time resolutions. Initially, concentrations from one-second (1 s) measurements (except O_3_) that fall below the limit of detection (LOD) were substituted with half the LOD of the monitored air quality features. The 8 s rolling median was aggregated from 1 s monitoring during the “drive pass” time window (Apte et al. 2017). Any 8 s rolling median below the LOD will be substituted with the corresponding 1/2 LOD value. The LODs for the monitored pollutants at 1 s and 8 s resolutions are listed in Table S1 of the SI. The statistical summary of the raw data for the 1 s and 8 s rolling median can be seen in Table S3 of the SI. The final dataset consists of 5.03 million 1 s measurements of PM_2.5_, NO_2_, NO, CO, and CO_2_, and 1.45 million of O_3_. Furthermore, we excluded any missing values for the key variables (e.g., 23.8–38.6%) (Table S3b) and retained 377,113 8 s measurements. The data was subsequently down-sampled to 1-h and daytime resolution to reduce the size of the dataset.

### Statistical analysis

In this study, PM_2.5_ concentrations are reported in the unit of µg/m^3^. NO_2_ and NO recorded in parts per billion volumes are converted to NOx, which is expressed as NO_2_ in the unit of µg/m^3^ in this study. We employed the Kolmogorov–Smirnov test to check central tendency and data distribution, and the median values were reported as core estimates. One-hour and daytime median values were derived from 8 s median aggregation. Instead of the daytime average, a daily 1-h maximum of O_3_ concentration was utilized in air quality evaluation. Correspondingly, subsampled data (e.g., high pollution days) was produced based on the 1-h and daytime resolution that showed concentrations exceeded the WHO AQGs and EU legal standards to explore the pollution episodes and hotspots. Statistical results were expressed in terms of interquartile range, including 1st, 5th, 25th, 75th, 95th, and 99th percentile. Data processing and statistical analysis were performed using the R statistical computing environment (https://www.r-project.org/), and *ggplot2* R package was used for data visualization.

### Baseline extraction

Atmospheric background and accumulated local emissions contribute to time series variation of air pollutant concentrations in urban area (Li et al. 2019b). In mobile monitoring, the background signal (e.g., low-frequency variations) is characterized by smooth variation at an hourly scale, while the rapid concentration spikes occur from seconds to minutes (Tan et al. 2014). In this study, we employed a baseline extraction method to divide the monitored concentrations $({C}_{t}(k,j)$) into the background and local emission concentrations using the following equations:

1 $${C}_{t}(k,j)={C}_{bg}(k,j)+{C}_{lc}(k,j)$$

2 $${C}_{bg}\left(k,j\right)={C}_{t, i}(k,j)$$

3 $${P}_{lc}(k,j)={C}_{lc}(k,j)/{C}_{t}(k,j)$$

where *C*_*lc*_(*k*, *j*) refers to local emission concentrations for pollutant *k* at time *j*, and *C*_*bg*_ (*k*, *j*) represents the background concentration for pollutant *k* at time *j*. The contribution from local emissions pollution $({P}_{lc}(k,j)$) is the ratio of ${C}_{lc}(k,j)/{C}_{t}(k,j)$.

The hypothesis proposes that the lowest percentile (${C}_{t, i}(k,j)$) signifies a background concentration characterized by a stable variation, which is minimally influenced by peaks originating from local sources (e.g., on-road pollution). The same method has been employed in previous studies to divide on-road background and accumulated vehicle emissions of the mobile monitoring air concentrations (Bukowiecki et al. 2002; Li et al. 2019b; Wei et al. 2021). In this study, we employed the 5th percentile for a “drive pass” time window (i.e., 8 s) to reconstruct a complete baseline to account for the background contribution to PM_2.5_ and NO_2_. Additionally, minima of 1-h median and daytime median were employed for the estimation of short-lived and longer-lived local pollution events.

### Optimized hotspot analysis

The identified highly polluted days that exceeded the WHO AQGs for 24-h levels of NO_2_ and PM_2.5_ were employed as input (Table 3) for optimized hotspot analysis. Both pooled data and single-day monitoring were included for analysis. In this study, the significant hot (i.e., significant spatial clusters of high values) and cold spots (i.e., low values) were calculated using the Getis-Ord Gi* statistics in Arc GIS Pro. We employed “count incidents within fishnet grid” method for aggregating datasets, while PM_2.5_ and NO_2_ concentrations were spatially joined in the analysis field. This approach has been utilized for hotspot analysis in prior research (Cummings et al. 2021). The tool aggregates the measured pollutants (e.g., points) and performs scale of analysis, and the outputs were corrected for both spatial dependence and multiple calculations. The outputs included a new feature class with a *z*-score, *p*-value, and significance level categories (Gi_Bin: 99% hot, 95% hot, 90% hot, not significant, 90% cold, 95% cold, and 99% cold).

## Results and discussion

### Summary statistics of monitored air pollutants

The current meta-data covers a total of 377,113 single-street segment (8 s) monitoring across Dublin. Table 1 shows descriptive statistics of mobile monitoring for PM_2.5_, NO_2_, O_3_, CO, and CO_2_. Specifically, the median NO_2_ concentration was 27.0 µg/m^3^ across single-segment measurements, and 95th of NO_2_ concentration reached 280 µg/m^3^. The median PM_2.5_ concentration was 6.6 µg/m^3^, with the 95th reaching 19.3 µg/m^3^. The median and 95th of measured CO_2_ were 442.3 ppm and 495.9 ppm. Median O_3_ and CO concentrations across the segments were 46.2 µg/m^3^ and 0.316 ppm, respectively, with 95th reaching 77.8 µg/m^3^ and 0.697 ppm. The mobile platform enables measurements with a high spatial coverage of traffic-related air pollutants at the hyperlocal level. The findings revealed that particle and gas pollutant concentrations exhibit significant dynamics across road segments. Previous studies indicated that spatiotemporal variability of air pollutants through mobile monitoring would be affected by various factors, including background matrix, meteorology, road traffic (e.g., speed limits, fleet composition), street topology, or pollution of spikes originating from nearby sources (Hofman et al. 2022a; Tang et al. 2020).

**Table 1:** Summary descriptive statistics of air pollutants from a single-street segment (8 s) from multiple measurements of mobile monitoring

	PM_2.5_ (µg/m^3^)	NO_2_ (µg/m^3^)	O_3_ (µg/m^3^)	CO (ppm)	CO_2_ (ppm)
Minimum	0.5	14.2	2.8	0.161	379.6
1st^a^	0.8	14.2	2.8	0.246	413.4
5th^b^	1.7	14.2	12.5	0.273	419.7
25th^b^	4.0	14.2	32.5	0.278	430.9
Median	6.6	27.0	46.2	0.316	442.3
Average	8.2	73.0	46.1	0.360	448.4
75th^b^	10.7	64.8	60.0	0.432	459.6
95th^b^	19.3	280	77.8	0.697	495.9
99th^b^	30.0	669	91.5	1.225	536.2
Maximum	781	4735	282	21.0	1413
*N*^c^	377,113	377,113	377,113	377,113	377,113

^a^1st percentile of the observation

^b^5th, 25th, 75th, 95th, 99th percentiles of the observation

^c^Data points for the 8 s rolling median from single segment drive pass

^d^NOx in µg/m^3^ is expressed as NO_2_. At 20 °C and 760 mmHg, a conversion factor was used, i.e., (NO ppb + NO2 ppb) * 1.9125 = NO_x_ µg/m^3^

As shown in Table 2, this mobile monitoring campaign is characterized by a high coverage density and measuring frequency for 1491 h on 233 sampling days. The 1-h median for NO_2_ varies from 14.2 to 470.4 µg/m^3^. The results in Table 2 showed that the EU legislation for 1-h NO_2_ limited values (i.e., 200 µg/m^3^) exceeded 6 h/year (0.4%). Some evidence suggests short-term exposure to a 1-h mean value of NO_2_ below 200 µg/m^3^ is associated with adverse health effects for patients with severe asthma (Latza et al. 2009). The daytime median of NO_2_ was 24.8 µg/m^3^, and 115 (49.4%) of the sampling days exceeded the 24-h WHO AQGs for NO_2_ (25 µg/m^3^) during the monitoring period. During daytime monitoring, PM_2.5_ concentrations vary from 1.4 to 29.5 µg/m^3^, with a median of 6.3 µg/m^3^. The results showed that the targeted WHO AQGs for 24 h PM_2.5_ (15 µg/m^3^) were exceeded in 21 days/year (9%). The WHO recommended to implement corrective actions to mitigate against PM_2.5_ pollution if the 24-h mean exceeded 3–4 days/year. The dominance of NO_2_ is reflected in the numbers of hours and days that pollutant concentrations exceeded the hourly and daily recommended concentrations. Tubridy et al. (2022) highlighted a breach of the EU limit value for NO_2_ and PM_2.5_ was evident in Dublin. These results were consistent with the findings from the Environmental Protection Agency (EPA), Ireland, which reported that NO_2_ is the primary pollutant from the transport sector in Dublin (Spohn et al. 2021), followed by PM_2.5_ and PM_10_ (Tang et al. 2019).

**Table 2:** Summary descriptive statistics of hourly and daytime concentrations for PM_2.5_ (µg/m^3^), NO_2_ (µg/m^3^), O_3_ (µg/m^3^), CO (ppm), and CO_2_ (ppm) from mobile monitoring

	Hourly					Daily^d^				
	PM_2.5_	NO_2_	O_3_	CO	CO_2_	PM_2.5_	NO_2_	O_3_	CO	CO_2_
Minimum	0.5	14.2	2.8	0.235	402.6	1.4	14.2	2.8	0.252	410.4
1st^a^	1.2	14.2	2.8	0.257	415.4	1.6	14.2	2.8	0.259	415.8
5th^b^	1.8	14.2	16.5	0.284	422.1	2.2	14.2	21.3	0.291	424.4
25th^b^	4.1	14.2	35.8	0.323	432.8	4.4	14.2	38.0	0.328	433.7
Average	7.6	32.3	47.2	0.371	447.2	7.6	28.4	48.0	0.362	444.1
SD^c^	5.0	29.6	17.9	0.071	21.3	4.7	15.7	16.0	0.054	16.3
Median	6.3	25.3	47.6	0.361	442.8	6.4	24.8	48.2	0.358	441.6
75th^b^	10.0	40.7	59.3	0.401	456.8	9.7	36.9	60.0	0.389	452.2
95th^b^	17.8	70.4	74.8	0.492	485.9	17.4	57.6	72.3	0.442	470.6
99th^b^	24.8	108.9	91.2	0.638	513.8	22.6	82.2	78.8	0.574	504.2
Maximum	32.2	470.4	110.8	0.824	636.3	29.5	94.6	91.3	0.643	543.8
*N*^e^	1491	1491	1491	1491	1491	233	233	233	233	233
Exceedance (%)^g^										
WHO 2021 AQGs	-^h^	-	-	-	-	15^f^ (9%)	25^f^ (49.4%)	100^f^ (0)	4 (0)	-
EU legal standards	-	200^f^ (0.4%)	-	-	-	-	-	120^f^ (0)	-	-

^a^1st percentile

^b^5th, 25th, 75th, 95th, 99th percentiles of the observations

^c^*SD* standard deviation

^d^Daily refers to the daytime statistics in this study

^e^Data points for the hourly and daytime statistics aggregated from 8 s median

^f^Refers to WHO AQGs and EU standards, respectively

^g^Percentage was calculated using the exceeding hourly or days divided by total sampling hours or days

^h^Not available

As shown in Table 2, the AQG levels (range from 100 to 120 µg/m^3^) did not exceed for 8-h mean O_3_ concentrations. The findings from the mobile monitoring, which recorded the average 8-h O_3_ concentration (48.0 µg/m^3^), align with previous results observed in Dublin at the urban site. McHugh et al. (2023) indicated that O_3_ concentrations in Dublin at urban sites were consistently lower than those in suburban or coastal areas. The WHO has no AQGs set for CO_2_ and concentrations of CO remained below the AQGs throughout the monitoring period.

### Time series of air pollutants

**Fig. 2:**
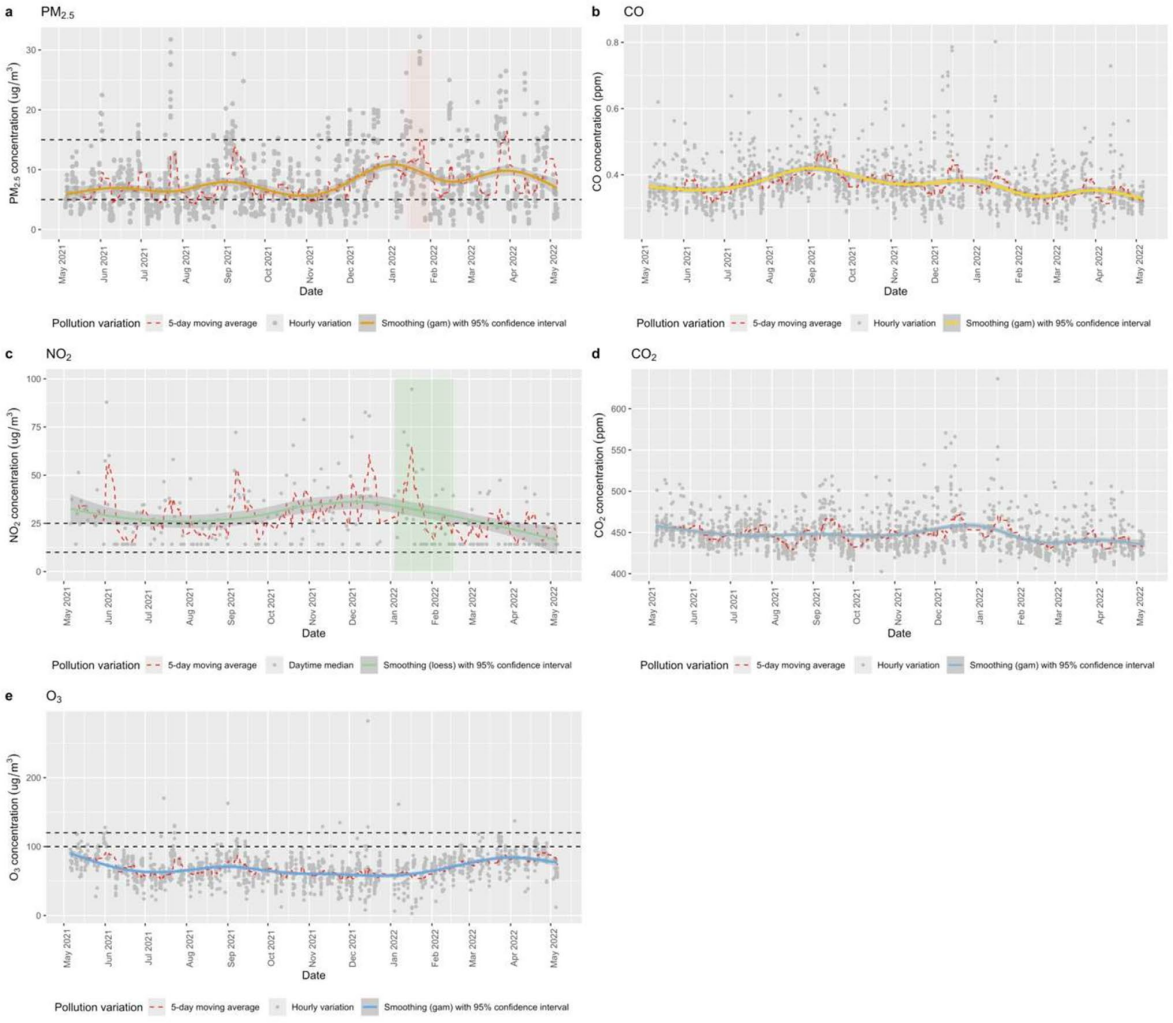
Time series of hourly **a** PM_2.5_, **b** CO, **c** NO_2_, **d** CO_2_, and **e** O_3_ from Google Air View vehicle during May 2021 and May 2022 in Dublin city. Hourly median concentrations on each day were used for calculation, except the O_3_ hourly maximum was utilized for calculation. Notes: The red dash lines show the 5-day moving average variations. The solid lines were drawn using the smoothing (gam, loess) trend function and the band indicates the confidence interval (0.95 by default). The black dashed lines indicate to the 24 h and annual of WHO AQGs for the specified pollutants

Figure 2 shows the temporal variability of PM_2.5_, CO, NO_2_, CO_2_, and O_3_ between May 2021 and May 2022. In addition to hourly and daytime variations, the 5-day moving average and data smoothing across the monitoring period are illustrated in Fig. 2. The smoothing pattern shows the PM_2.5_ concentration exceeded the WHO AQGs for an annual value of 5 µg/m^3^. Furthermore, the smoothing trend highlights a rise in concentrations observed during the winter season. The monthly variations of the monitored pollutants are presented in Fig. S1. PM_2.5_ concentrations were lower from May to August, experiencing a steady increase in December and January. These findings align with results obtained from fixed-site monitoring at community sites in Dublin. For example, Lee et al. (2022) found that the ambient PM_2.5_ concentrations (9–13 µg/m^3^) in Dublin surpassed the WHO AQGs with significant seasonal variability showing higher PM_2.5_ levels in December, which could be attributed to solid fuel burning for domestic heating in the winter season.

As can be seen in Fig. 2, the measured NO_2_ is highly dynamic during the on-road mobile monitoring. The 5-day moving average shows a higher frequency of elevated NO_2_ pollution events compared to PM_2.5_. The smoothing pattern demonstrated the NO_2_ concentrations were above the WHO AQGs for an annual value of 10 µg/m^3^. Much greater monthly variation is evident for NO_2_ than PM_2.5_ (Fig. S1). There was moderate evidence showing that short-term exposure to a 24-h mean NO_2_ value of ~ 50 µg/m^3^ increased respiratory hospital admission and mortality (Quintyne et al. 2021). The CO and CO_2_ concentrations fluctuate slightly across seasons. In general, the 5-day moving average shows similar peaks and valleys occurred across measured pollutants except O_3_. For the 8-h average O_3_, an inverse trend was shown compared to NO_2_, with the maximum concentrations in April and May, while minimum values were shown in November and December.

The potential relationships between the mobile monitored pollutants were investigated using Spearman’s correlation coefficients, and results are shown at temporal variations from 1-h and daytime levels in Fig. S2. Weak correlations were found for 1-h and daytime median PM_2.5_ with CO_2_ (*r*_s_ = 0.26), CO (*r*_s_ = 0.25), and NO_2_ (*r*_s_ = 0.27–0.30), most likely due to the varied sources of PM_2.5_ emission in Dublin. A report from EPA Ireland indicated major sources of ultra-fine particles in Dublin were secondary pollutants and oil, traffic, solid fuels, and cooking (Ovadnevaite et al. 2021). Moderate to strong correlations were found for NO_2_ with CO (*r*_s_ = 0.48–0.67) and CO_2_ (*r*_s_ = 0.47–0.74), suggesting that they share similar emissions from the transport sector (Ghahramani and Pilla 2021). In contrast, negative correlations were shown for O_3_ with CO_2_, CO, and NO_2_. This is expected since O_3_ is a secondary pollutant, with NO_2_ serving as its precursor. There was no notable weekday variability observed for the monitored pollutants (Fig. S3). In contrast, other studies have reported varying results, such as Martínez Torres et al. (2020) found that NO_2_ concentrations were higher from Wednesday to Friday compared to other weekdays in Dublin. Weekday-weekend variation is not the primary focus of this study and has been discussed in previous articles. For example, Lee et al. (2022) suggested that weekday PM_2.5_ concentrations were higher than weekends in Dublin.

### Diurnal variation of air pollutants

**Fig. 3:**
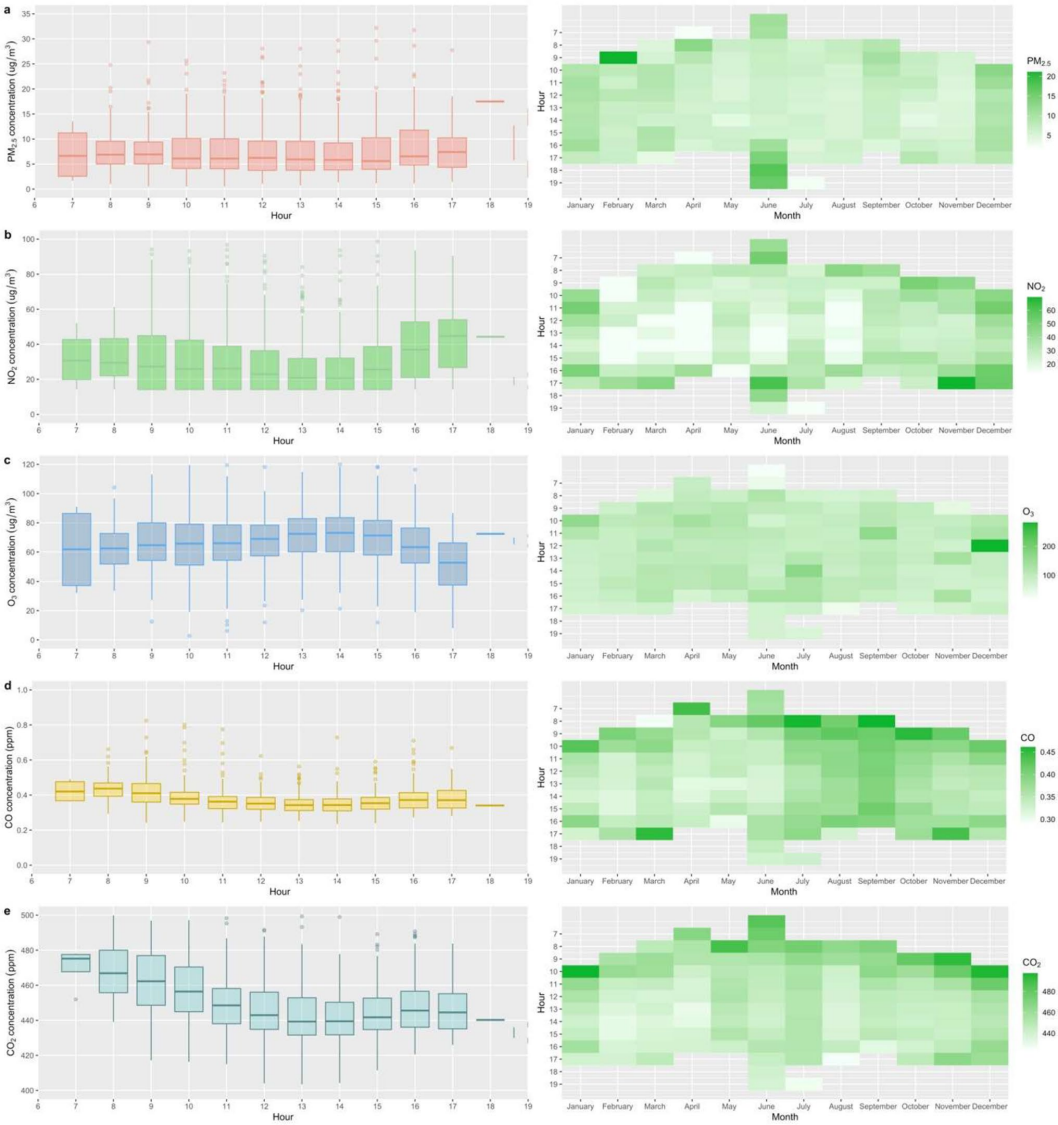
Diurnal trends (left) and heatmap (right) of **a** PM_2.5_, **b** NO_2_, **c** O_3_, **d** CO, and **e** CO_2_ by Google Air View vehicle in Dublin city (*n* = 1486 h). Box plots represent the 25th and 75th percentiles, with the median (50th percentile) represented by the horizontal black line. Whiskers represent the lowest and largest values of 1.5*IQR

Figure 3 illustrates the boxplots of hourly variation of the mobile monitored PM_2.5_, NO_2_, O_3_, CO, and CO_2_, respectively, during daytime (mainly from 7:00 to 17:00) between May 2021 to May 2022. One-hour medians are utilized to characterize the diurnal variation. In terms of temporal evolution, although mobile monitoring was conducted from different postal areas and across various seasons in Dublin, the basic trend illustrates elevated levels of PM_2.5_ in the early morning (8:00–9:00) and in the early evening around 17:00, respectively, during weekdays (Fig. 3). Figure S4 shows the diurnal variation in average PM_2.5_ from ambient locations characterized by urban background, suburban background, and urban traffic. Peak ambient PM_2.5_ values occurred at 20:00–21:00, showing higher night-time concentrations than daytime. These elevated levels are most likely attributed to regional transportation emission from residential solid combustion. These findings align with the results from air pollution monitoring conducted outside three maternity hospitals in Dublin. The study reported a similar daytime pattern for PM_2.5_ and showed higher night-time PM_2.5_ concentrations than daytime levels (Lee et al. 2022). Additionally, the results indicated these high concentration events for PM_2.5_ occurred mainly during working hours on weekdays in December.

Hourly averages of NO_2_ exhibited a distinct daytime variation trend, with peaks observed at 7:00–8:00 in the morning and 17:00–18:00 in the early evening. Martínez Torres et al. (2020) found a similar NO_2_ diurnal pattern in Dublin, showing the highest NO_2_ concentration at 8:00–9:00 and 17:00–18:00. These findings indicate that attribution of the direct impact of traffic emission on NO_2_ is more evident than for PM_2.5_, suggesting NO_2_ serves a better indicator for characterizing traffic-related air pollution in both short-term and long-term. These patterns coincided with the morning rush hour and traffic hour after work, and the valley values were observed at noon. A previous study revealed that the average hourly traffic achieved the peak value during the morning (8:00 am) and afternoon traffic (4:00–5:00 pm) in Dublin city (Tang et al. 2019). Moreover, the time and distribution pattern of the peaks shifts with the seasons, with the morning peaks in June occurring at 7:00–8:00. Evening peaks for NO_2_ concentrations mostly occurred at 16:00 in December and January. Previous findings in the UK suggested a distinct shift in peak timing to earlier evenings in the winter season (Wyche et al. 2020). Figure S3 shows that ambient NO_2_ levels gradually increased from 5:00 am, and the morning peaks occurred at about 8:00 in the morning at the urban traffic site and 9:00 at the urban and suburban background sites. The evening peaks occurred at 18:00 at the traffic site and shifted to 19:00–21:00 at the urban and suburban background sites. These results highlight the impact of non-traffic emissions.

O_3_ exhibited a contrasting diurnal trend compared to PM_2.5_ and NO_2_, and the highest O_3_ concentrations were observed in the afternoon (13:00–15:00). This could be due to photochemical effects that increase O_3_ concentrations during the daytime. The diurnal pattern for O_3_ is consistent with previous findings from ambient sites in Dublin (Tripathi et al. 2012) and modeling results (Donnelly et al. 2019). The O_3_ concentrations from mobile monitoring exhibited consistently lower concentrations than other suburban or background areas. A previous study was conducted in Dublin to investigate the spatial–temporal variation of traffic and urban monitoring sites (Perillo et al. 2022), and the results demonstrated reductions in PM_2.5_ and NO_2_ concentrations and an increase in O_3_ concentrations during COVID restrictions. Diesel vehicles are prominent sources of NO_2_ and PM_2.5_ (Tang et al. 2019). The CO diurnal profiles showed a similar but less clear pattern than NO_2_, which reflects the diesel-powered vehicle fleet. As for CO_2_, higher concentrations were mostly shown in the early morning.

### Partitioning pollution into the background and local contribution

The total pollutants time series from mobile monitoring were divided into background and locally emitted contributions. In this study, we chose the 5th percentile of 1 h (Table S5), minima over 1-min, 1-h, and daytime data as background at different time resolutions. Compared to the 5th percentile of the whole dataset, the corresponding averages of minima for 1-h NO_2_ deviated by about 5%, while they doubled for PM_2.5_. The development of the 5th percentile curve for PM_2.5_ and NO_2_ shows a consistent background trend compared to the minima during the entire monitoring period (Fig. S5). Similarly, strong correlations were shown for PM_2.5_ from the estimated background (1-h minimum) and those measured at the urban background site (*r*_s_ = 0.76, *p* < 0.01). Based on these results, the uses of minima are considered valid to obtain an estimate of the local contribution at different time resolutions where mobile monitoring was conducted. Furthermore, as suggested by Zimmerman et al. (2020) and Hofman et al. (2022b), a change on the order of 1 h is considered a short-lived event, and a longer-lived event is defined as occurring on a time scale of daytime basis (e.g., 2–8 h).

**Fig. 4:**
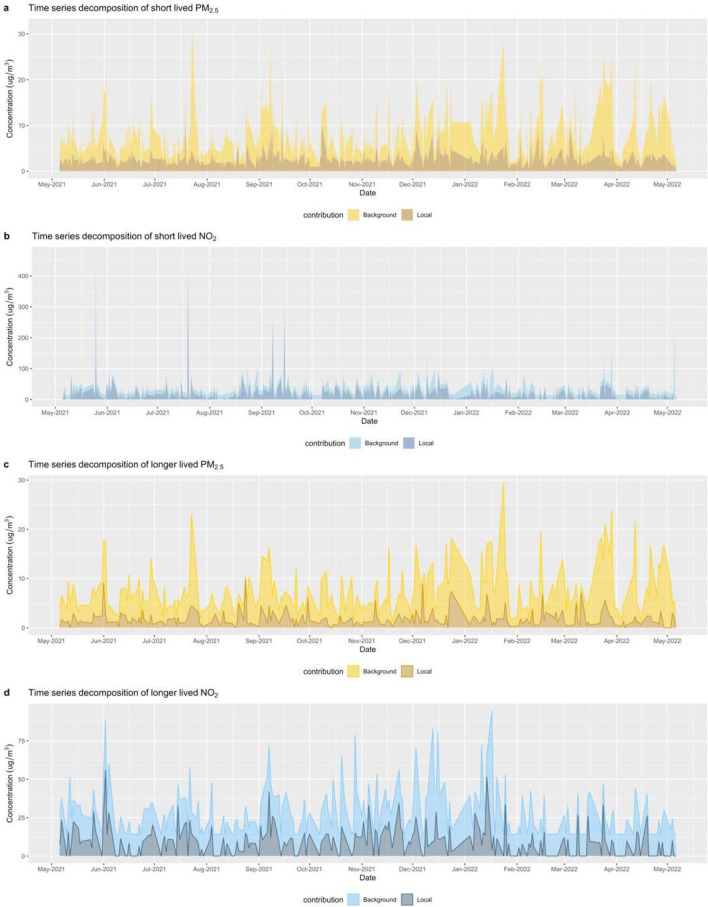
Time series decomposition of background and local contribution for PM_2.5_ and NO_2_. Notes: **a** and **b** refer to short-lived events for PM_2.5_ and NO_2_ (i.e., 1 h), and **c** and **d** refer to longer-lived events for PM_2.5_ and NO_2_ (i.e., ~ 8 h)

Figure 4 shows the division of total pollution into background contributions alongside short-lived and longer-lived events for PM_2.5_ and NO_2_. Of the total enhancement above background, the short-lived event enhancement (1 min to 1 h) accounts for 20.8–42.2% and 36.0–40.6% of the total concentration for PM_2.5_ (Fig. 4a) and NO_2_ (Fig. 4b). For short-lived events, NO_2_ exhibits sharper peaks than PM_2.5_. These results indicate the general public is at potentially higher risk during evening rush hours when background and local traffic emissions are tremendous. As can be seen in Fig. 4c and d, the longer-lived events (i.e., daytime) account for about 25% of the total concentration for both pollutants. Furthermore, previous findings suggested there are currently severe issues associated with air pollution in Dublin arising from both the transport sector and the use of solid fuel for domestic heating (Ovadnevaite et al. 2021). As for NO_2_, vehicle traffic is the main source along with electricity generation stations and industry. NO_2_ levels fluctuate more than PM_2.5_ during daylight hours at the local level.

**Fig. 5:**
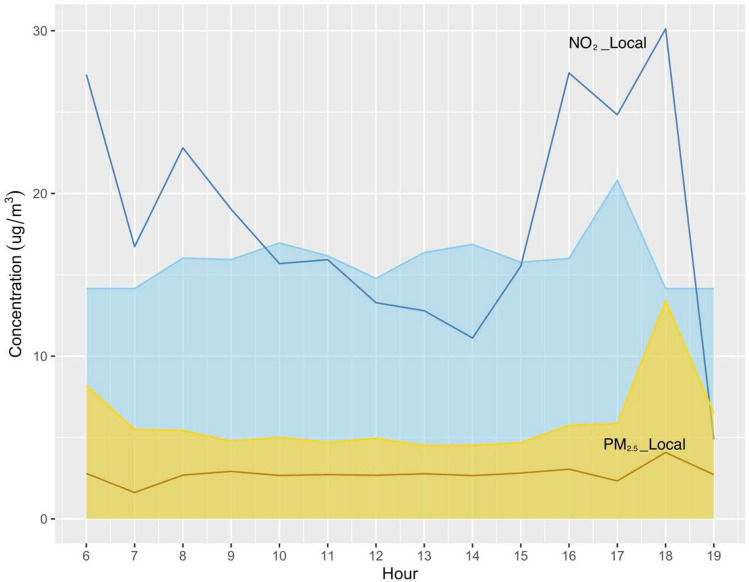
Diurnal variation in background and short-period emission of PM_2.5_ and NO_2_ during the entire study period. Notes: Lines represent short-lived events and areas represent background concentrations; the golden color refers to PM_2.5_ and the blue color refers to NO_2_

The decomposition results for the highly polluted days with the background and local contributions are illustrated in Fig. S6. Of particular interest are the pollution episodes that occurred on 17th January and 29 March 2022, revealing decomposed background concentration of NO_2_ and PM_2.5_ reaching 53.6–77.8 µg/m^3^ and 13.0–20.4 µg/m^3^, which were mainly due to background contribution, could be regional transported sources. During these regional pollution events, high concentrations of NO_2_ and PM_2.5_ were observed at urban and suburban background ambient sites. Subsequently, we compared the diurnal variability of PM_2.5_ and NO_2_ to illustrate the temporal trend and potential contributions from local and background sources during highly polluted days. As shown in Fig. 5, for short-lived events, traffic emissions contribute to both PM_2.5_ and NO_2_. The results show the diurnal trends are evident in NO_2_ concentration in the short term. The mean NO_2_ concentration during traffic peak time is a factor of 2 to the background and a factor of 2–3 to the off-peak. These results are consistent with previous findings from Zimmerman et al. (2020), which suggested near road concentrations of PM_2.5_ were only moderately elevated, while NO_2_ has much higher near roadway concentration enhanced (> 3–5 times). The probability of experiencing adverse health responses could be greatest when the concentration of NO_2_ is elevated. During polluted days, local NO_2_ has two peaks associated with morning (8:00) and early evening (16:00–18:00) rush hour, contributing to 54.4–68.0% of total NO_2_ concentrations. This means that the monitored NO_2_ is significantly enriched mainly from local traffic emissions. Additionally, there were peaks around 17:00–18:00 for background PM_2.5_ and NO_2_; this may suggest that their temporal variations are influenced by non-traffic sources. Overall, NO_2_ had the larger temporal variations (33.2%), whereas PM_2.5_ was less variable (22.3%). This is consistent with the traffic site monitoring results as shown in Fig. S3. Song et al. (2021) suggested diesel vehicles have larger NOx emission factors than gasoline vehicles. These findings highlighted the influence of diesel traffic on elevated NO_2_ levels in Dublin.

Figure S7 enables a comparison of the time series variability of PM_2.5_ and NO_2_ from mobile monitoring and ambient air quality monitoring stations (AQMS) on the same timescale. The time series variability of ambient and mobile results exhibited similarities but showed significant differences in concentrations. For example, mobile results were significantly higher than the traffic monitoring site for PM_2.5_ (7.2 µg/m^3^, *p* < 0.01), but less for NO_2_ (41.1 µg/m^3^) (Table S3). A total of 20.2% of the monitoring days showed higher mobile NO_2_ concentration than the corresponding traffic site measurement. As expected, PM_2.5_ is spatially homogeneously distributed across the city, and higher associations were found for mobile monitoring with AQMS measurement of PM_2.5_ (*r*_s_ = 0.72–0.80) than for NO_2_ (*r*_s_ = 0.33–0.37) (Table S4). Carthy et al. (2020) found that the Dublin traffic zone had the highest NO_2_ levels compared to other areas. A study by Li et al. (2019a) highlights that mobile measurement represents the urban stationary monitors within a 1 km^2^ area. These findings indicated that mobile monitoring exhibited a high variability compared to air quality monitoring stations, emphasizing the need for extensive spatial coverage to capture the variability of NO_2_ from traffic emission.

### Hotspots of traffic-related air pollutants

**Fig. 6:**
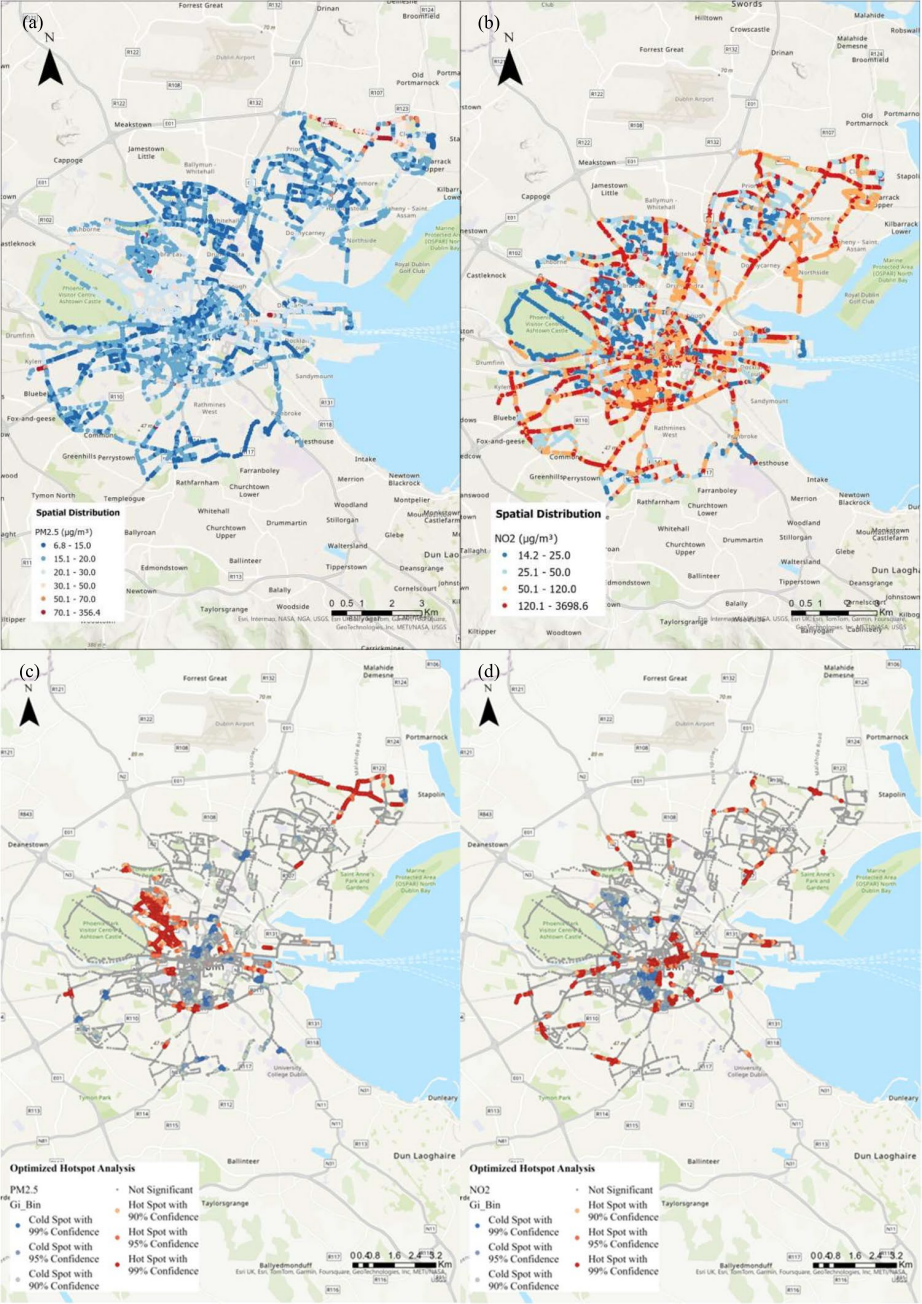
Spatial distribution of highly polluted **a** PM_2.5_ and **b** NO_2_ along with optimized hotspots analysis results for **c** PM_2.5_ and **d** NO_2_ in Dublin

By ranking the most polluted days along with sampling routes at the community level from high to low, Table 3 shows the details of several cases in which daytime NO_2_ and PM_2.5_ exceeded the WHO guidelines of 25 µg/m^3^ and 15 µg/m^3^ throughout the monitoring period. Mapping of the elevated NO_2_ and PM_2.5_ concentrations for these days is displayed in Fig. 6, and pollution hotspots were identified for PM_2.5_ and NO_2_. Table 3 outlines the hotspots description for PM_2.5_ corresponding to optimal hotspots. For example, high values of PM_2.5_ cluster are observed at highly trafficked areas in Dublin, notably along National Roads such as N1 at O’Connell Street Upper, N2 near Grangegorman Upper, N3 at Dalymount, and N4 at Saint John’s Road West. In addition, Regional Roads like R101 at Cork Street and R109 from Parkgate to Chesterfield venue were PM_2.5_ hotspots with 90 to 99% confidence. High concentrations of PM_2.5_ clusters are also linked to industrial activities, for example, vehicle traffic at the landside of Dublin Port (Djordjević et al. 2023), and emissions from nearby restaurants. Our findings in Fig. 6 further indicated that hotspots of NO_2_ are more dispersedly distributed than those of PM_2.5_. To compare the results for pooled data, Fig. S8 shows consolidated information derived from single-day output. The location and timing of hotspots varied from day to day, and the recurrent hotspots in specific locations suggest that there are areas in Dublin where traffic-related pollutant concentrations are consistently elevated relative to the surrounding area. A recent publication indicates a signification association between urban green space and decreased NO_2_ concentrations in Dublin (Sabedotti et al. 2023). Identification of hotspots for traffic-related pollution provides potential policy instruments from an urban planning perspective to prioritize urban public transit, walking, and cycling networks within the city. Establishing motorized traffic-free zones and expanding public green spaces would also contribute to reducing the PM_2.5_ and NO_2_ levels.

**Table 3:** Description of identified highly polluted daytime median of PM_2.5_ and NO_2_ and elevated concentrations

ID	NO_2_ (µg/m^3^)	PM_2.5_ (µg/m^3^)	Date of events^a^	Elevated concentration for NO_2_ (µg/m^3^)	Elevated concentration for PM_2.5_ (µg/m^3^)	Hotspots description for PM_2.5_
1	94.6	17.6	2022/01/17	22.5 ± 6.6	4.7 ± 0.8	Along R101 Cork Street, extending from Brabazon Row to Newmarket
2	87.9	17.8	2021/06/02	62.9 ± 30.5	4.6 ± 1.1	N4 Saint John’s Road West; from Custom House Quay, running along North Wall Quay to Cardiff Lane
3	72.4	15.5	2022/01/11	37.5 ± 9.2	6.0 ± 2.1	O’Connell Street Upper; from Promenade Rode, crossing Bond Drive to Tolka Quay Road and Alexandra Road (Dublin Port)
4	72.2	16.4	2021/09/07	46.7 ± 19.3	4.3 ± 1.3	Road along River Liffey extends to Temple Bar, crosses O’Connell Bridge and continues to the North wall
5	69.9	16.7	2021/12/03	48.0 ± 10.5	5.5 ± 1.6	N81 along the wood quay and extends to Harold’s crossroad; James’s street
6	65.5	17.1	2022/01/14	35.6 ± 17.3	6.5 ± 3.4	Crossroad around Newtown Court way-Clarehall Avenue and Malahide road-Churchwell drive
7	57.4	17.7	2021/06/01	33.0 ± 18.4	4.3 ± 2.6	The vicinity area encompassing Sir John Rogerson’s Quay and Hanover Quay
8	43.4	21.6	2022/04/12	30.1 ± 9.7	2.9 ± 1.1	From Custom House Quay, extends along North Wall Quay to Alexandra Road
9	36.6	16.4	2021/11/17	20.6 ± 13.7	4.0 ± 0.8	R101 Cork Street following Brabazon Row to Newmarket
10	34.6	17.3	2021/12/21	22.9 ± 7.1	4.8 ± 1.6	Residential area located at the southeast side of Riverston Abbey; intersection of R101 and N3 (Dalymount), extends along R101 to Chesterfield and North Road of Phoenix Park
11	29.8	16.7	2022/04/29	15.6	3.6	N.A. ^b^
12	28.6	23.1	2021/07/23	18.4 ± 13.6	4.6 ± 0.8	Stoneybatter and residential area situated at the East side of Riverston Abbey
13	25.9	23.9	2022/03/29	17.9 ± 15.5	3.2 ± 2.1	R109 stretches from Parkgate to Chesterfield venue; the triangle area extends from Grangegorman Upper along R101 to N2, passing the King’s Inns Court

^a^Daytime median > 15 µg/m^3^ for PM_2.5_ and > 25 µg/m^3^ for NO_2_

^b^*N.A.* not available

### Limitation of the study

While our study provided comprehensive insights into Google Air metadata, it is limited by the lack of weekend and nighttime monitoring of traffic-related air pollutants, which hinders a thorough assessment of diurnal variations and the analysis of meteorological effects on traffic-related pollution. The varied monitoring time of each segment could affect the spatial and temporal assessment of the measured pollutants. Our assessment of the decomposition of short-lived events did not consider the traffic fleet composition or street topology. There were no supporting data to explore the origin of pollution sources about the contribution from longer-lived events. Further research could explore the integration of regional pollution sources to develop targeted PM_2.5_ and NO_2_ mitigation policies aimed at addressing traffic pollution in Dublin.

## Conclusions

In this study, we demonstrate the potential for a mobile monitoring approach to investigate the hyperlocal distribution of air pollutants in Dublin, Ireland. This investigation characterizes traffic-related gas and particle pollutants after the COVID pandemic and provides an illustration of temporal variability, hotspot characterization, and local and background concentration contributions for mobile monitored PM_2.5_ and NO_2_. Median concentrations of daytime NO_2_ and PM_2.5_ exceeded the WHO 24-h AQGs for 49.4% and 9% of the sampling days throughout the monitoring period. The diurnal pattern was more evident for NO_2_ than PM_2.5_, with peak values occurring at 8:00 and 17:00–18:00. Seasonally, PM_2.5_ and NO_2_ concentrations are higher in winter than in the summer season, while no clear weekday variations were shown. Our findings demonstrate that elevated traffic-related NO_2_ in Dublin exhibited significant diurnal and spatial variations. Detailed knowledge of background and local contribution to mobile monitoring is important for highly polluted regions. This meta-data can be further implied for the sensitive analysis of the representativeness of mobile measurements and to obtain robust modeling prediction in space and time.

We can draw several conclusions by comparing the decomposed concentration and ambient data, NO_2_ is more affected by traffic emissions. Our data highlight the importance of non-traffic sources for temporal variation in the early evening. While traffic is a dominant source of NO_2_, non-traffic sources appear to be the dominant source for PM_2.5_. The importance of non-traffic sources suggests that merely regulating vehicle emissions may be insufficient to reduce PM_2.5_ concentrations to meet the new WHO guidelines. The findings highlight hyperlocal air monitoring is a complementary tool to provide meta-data for the research community. It enhances understanding of the implications of air quality policies and the effectiveness of air pollution mitigation plans from multiple emission sources. Furthermore, it enables providing air quality assessment to local environmental departments and policymakers for developing targeted strategies for mitigating traffic-related air pollution at a localized level.

## Supplementary Information

Below is the link to the electronic supplementary material.

Supplementary file1 (DOCX 18.0 KB)

## Declarations

### Citation of data

Google (2023) “Dublin_20210506_20220805_GoogleAclimaAQ” (https://data.smartdublin.ie/dataset/google-airview-data-dublin-city).
