# Supplementary material for: Evaluating background and local contributions and identifying traffic-related pollutant hotspots: insights from Google Air View mobile monitoring in Dublin, Ireland
Source: Environ Sci Pollut Res Int. 2024 Sep 10;31(44):56114–29. doi: 10.1007/s11356-024-34903-5 (PMC11420298; doi:10.1007/s11356-024-34903-5)
Supplement: Supplementary file 1 — Supplementary file1 (DOCX 18.0 KB) [file 11356_2024_34903_MOESM1_ESM.docx]

**Supporting information**

**Evaluating background and local contributions and identifying traffic-related pollutant hotspots: insights from Google Air View mobile monitoring in Dublin, Ireland**

Jiayao Chen^1^, Anna Molter^1^, José P. Gómez Barrón^1^, David O’Connor^2^, Francesco Pilla^1^

^1^ School of Architecture, Planning and Environmental Policy, University College Dublin, Dublin, Ireland

^2^ School of Chemical Sciences, Dublin City University, Dublin, Ireland

**Fig. S1**. Monthly variation of (a) PM_2.5_, b (CO), (c) NO_2_, (d) CO_2_, (e) O_3_, and (f) one-hour maximum O_3_ concentrations monitored from Google Airview vehicle. Notes: Median values were illustrated.

**Fig. S2**. Spearman’s correlation matrix of mobile monitored pollutants, a. hourly, and b. daytime.

**Fig. S3**. Weekday variations of (a) PM_2.5_, b (CO), (c) NO_2_, (d) CO_2_, (e) O_3,_ and (f) one-hour Maximum O_3_ concentrations.

**Fig. S4**. Hourly average diurnal variations of PM_2.5_ and NO_2_ concentrations at urban background (Rathmines), suburban background (Dun Laoghaire), and urban traffic sites (St. Johns). Notes: Dot lines for NO_2_ (Traffic dominated: StJohns) and O_3_ (Rathmines) were plotted on the secondary axis.

**Fig. S5**. Time series of estimated background (5^th^ percentile) and monitored concentration for PM_2.5_ (gray line) and NO_2_ (orange line). Notes: 5^th^ of the whole dataset was used for the decomposition. Black line refers to background NO_2_, and red line is the background PM_2.5_.

**Fig. S6**. Decomposition of background and local events for (a) PM_2.5_ and (b) NO_2_ during the highly polluted days.

**Fig. S7**. Time series of PM_2.5_ and NO_2_ from ambient monitoring sites along with daytime mobile monitoring results.

**Fig. S8.** Spatial distribution of high pollution levels for (a) PM_2.5_ and NO_2_ together with optimized hotspot analysis for (b) PM_2.5_ and (c) NO_2_ in Dublin. Note: Consolidated information derived from single day outputs were illustrated.

**
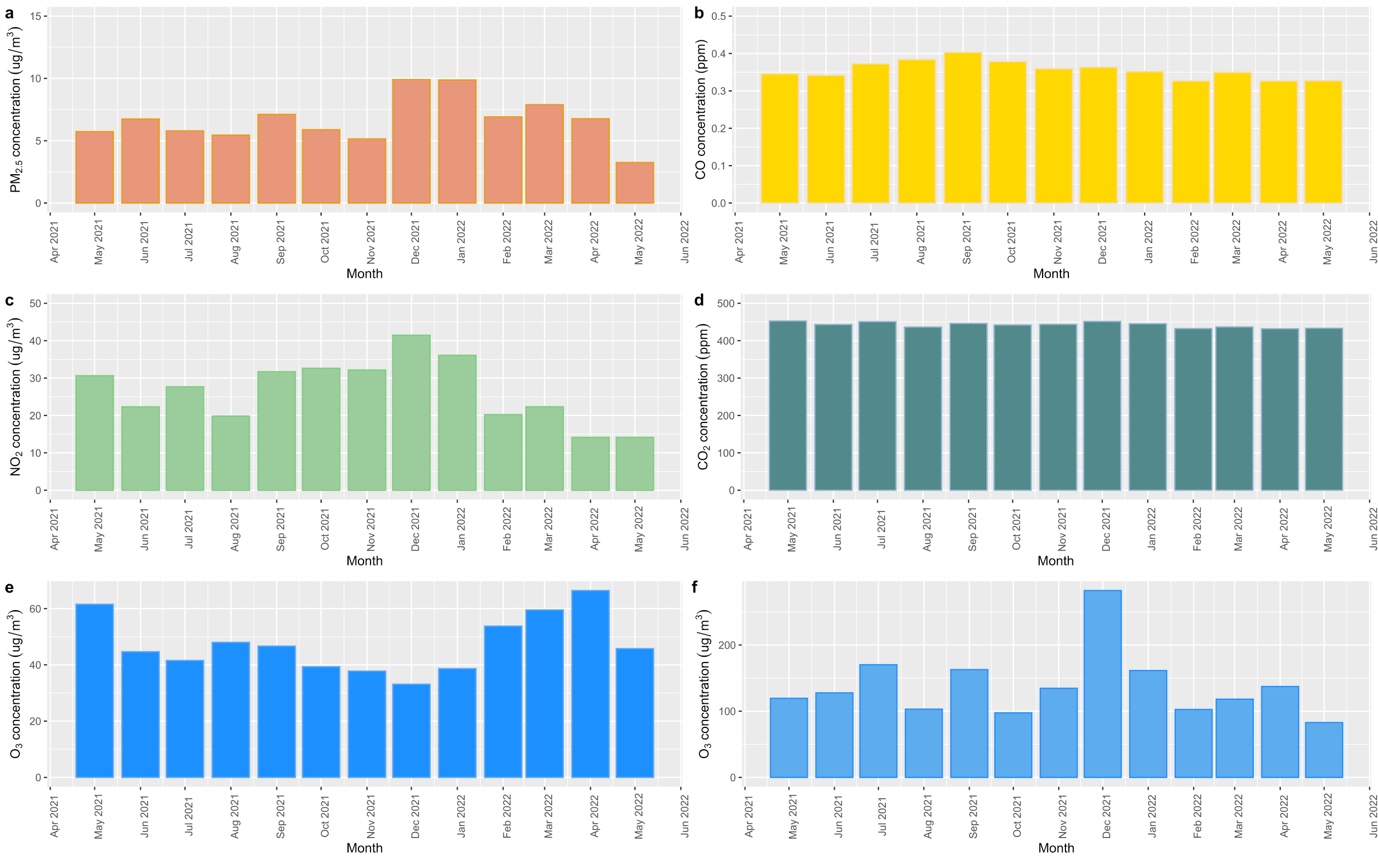
**

**Fig. S1**. Monthly variation of (a) PM_2.5_, b (CO), (c) NO_2_, (d) CO_2_, (e) O_3_, and (f) one-hour maximum O_3_ concentrations monitored from Google Airview vehicle. Notes: Median values were illustrated.


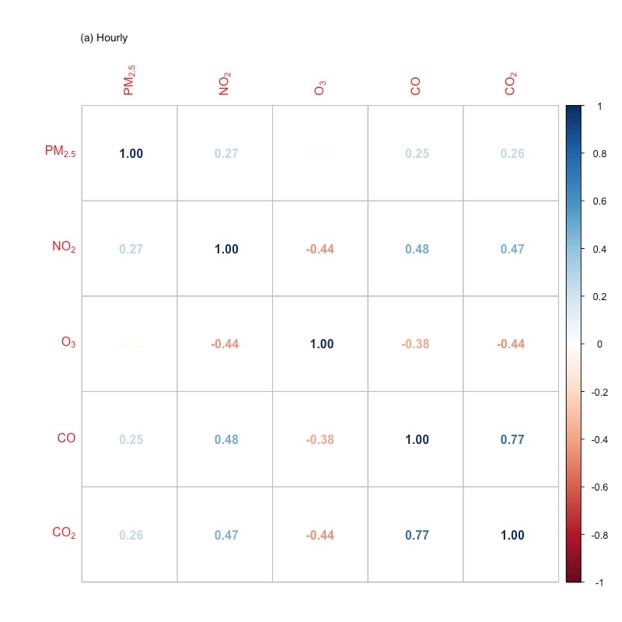

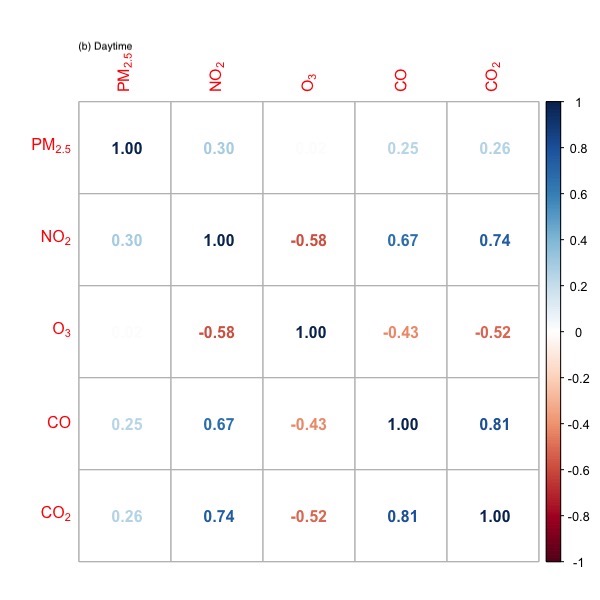


**Fig. S2**. Spearman’s correlation matrix of mobile monitored pollutants, a. Hourly, and b. Daytime.


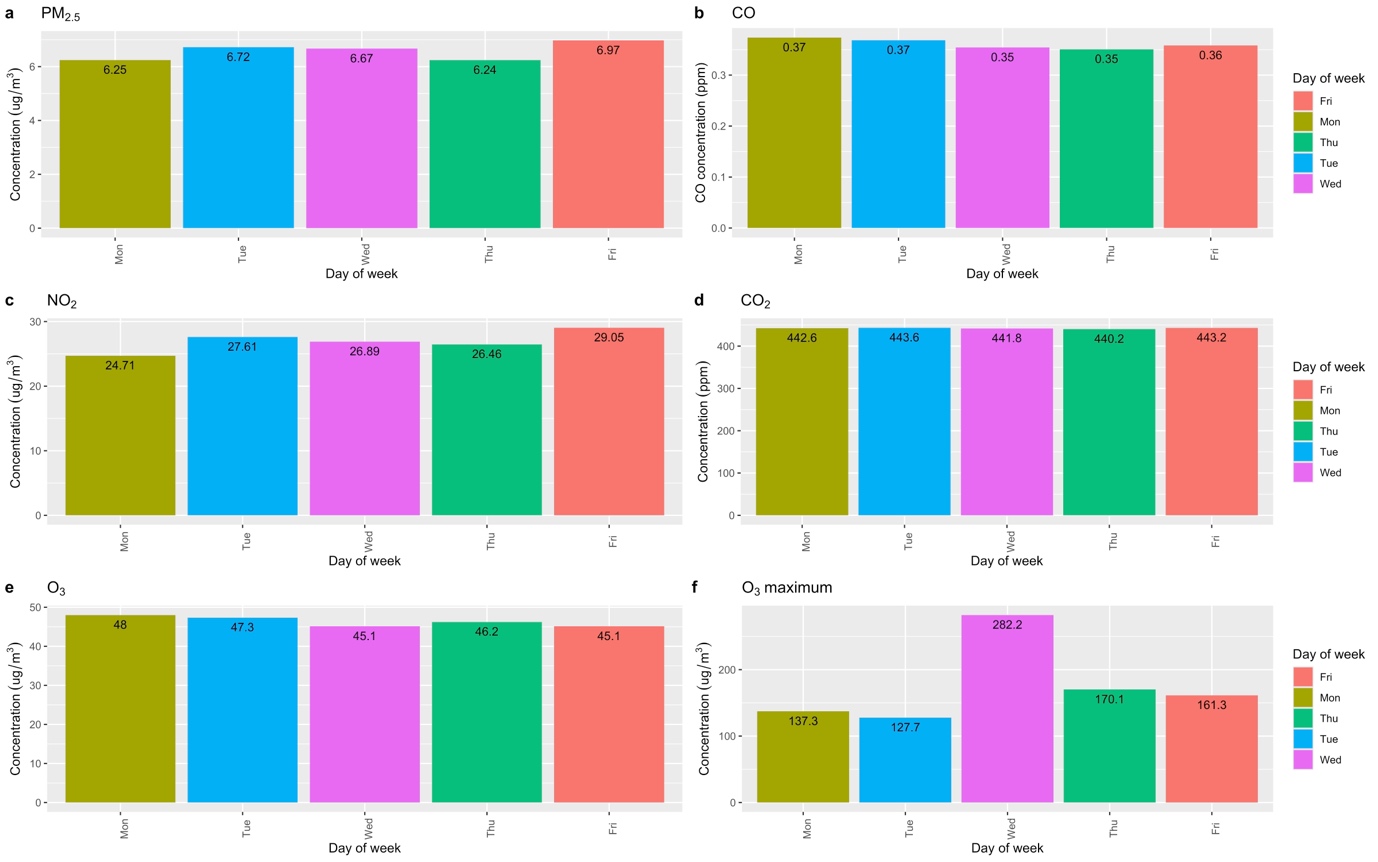


**Fig. S3**. Weekday variations of (a) PM_2.5_, b (CO), (c) NO_2_, (d) CO_2_, (e) O_3,_ and (f) one-hour maximum O_3_ concentrations.

**Fig. S4**. Hourly average diurnal variations of PM_2.5_ and NO_2_ concentrations at urban background (Rathmines), suburban background (Dun Laoghaire), and urban traffic sites (St. Johns). Notes: Dot lines for NO_2_ (Traffic dominated: StJohns) and O_3_ (Rathmines) were plotted on the secondary axis.


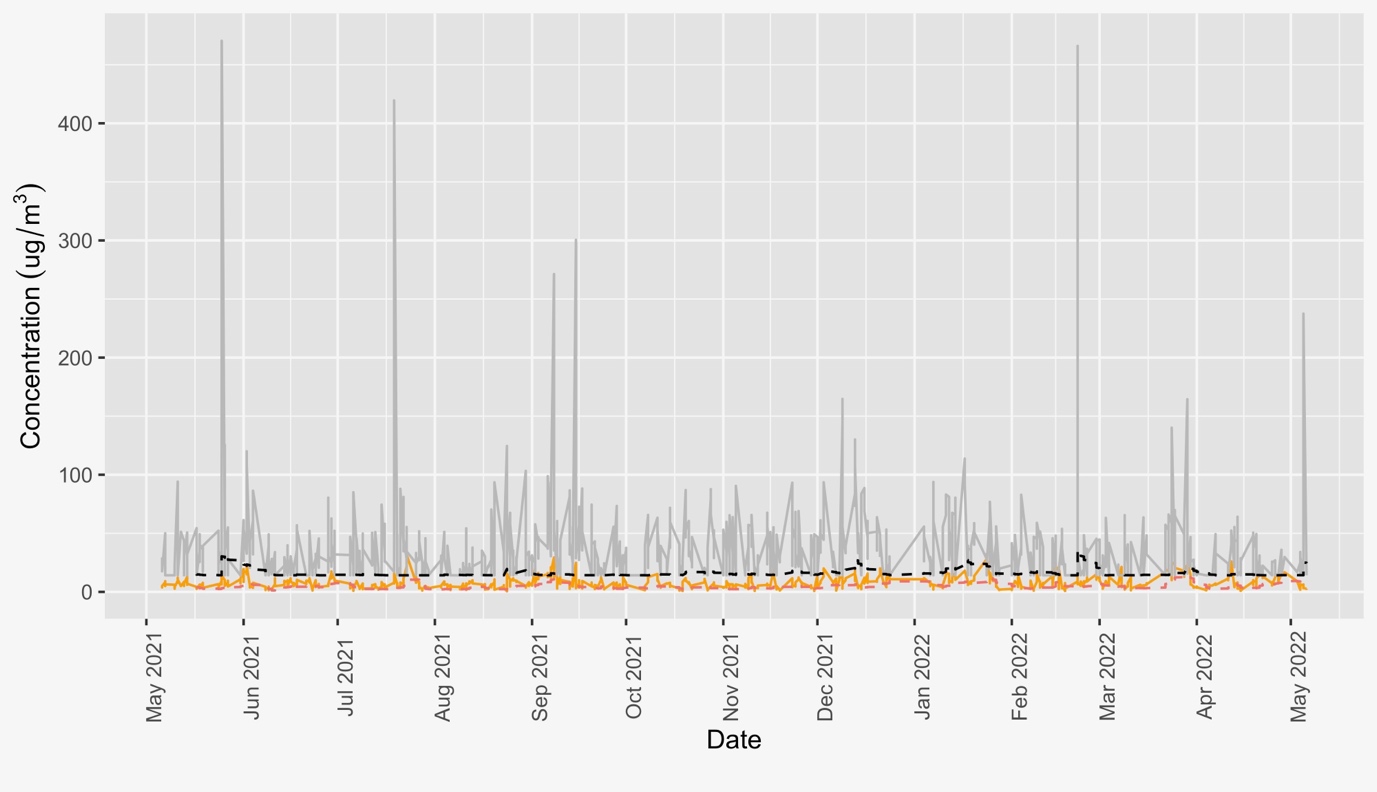


**Fig. S5**. Time series of estimated background (5^th^ percentile) and monitored concentration for PM_2.5_ (gray line) and NO_2_ (orange line). Notes: 5^th^ of the whole dataset was used for the decomposition. Black line refers to background NO_2_, and red line is the background PM_2.5_.

**Fig. S6**. Decomposition of background and local events for (a) PM_2.5_ and (b) NO_2_ during the highly polluted days.

**Fig. S7**. Time series of PM_2.5_ and NO_2_ from ambient monitoring sites along with daytime mobile monitoring results.

(a)

**
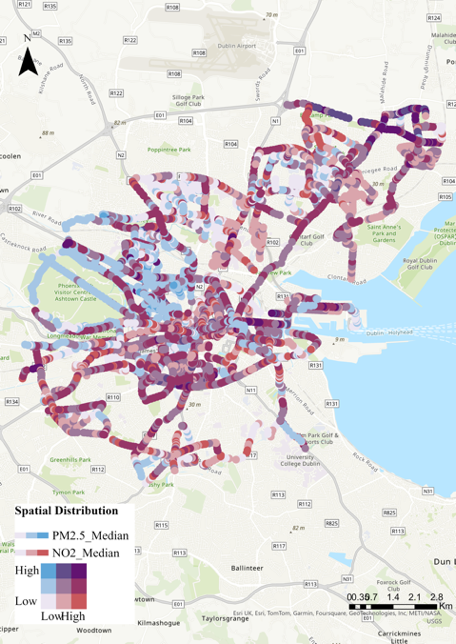
**
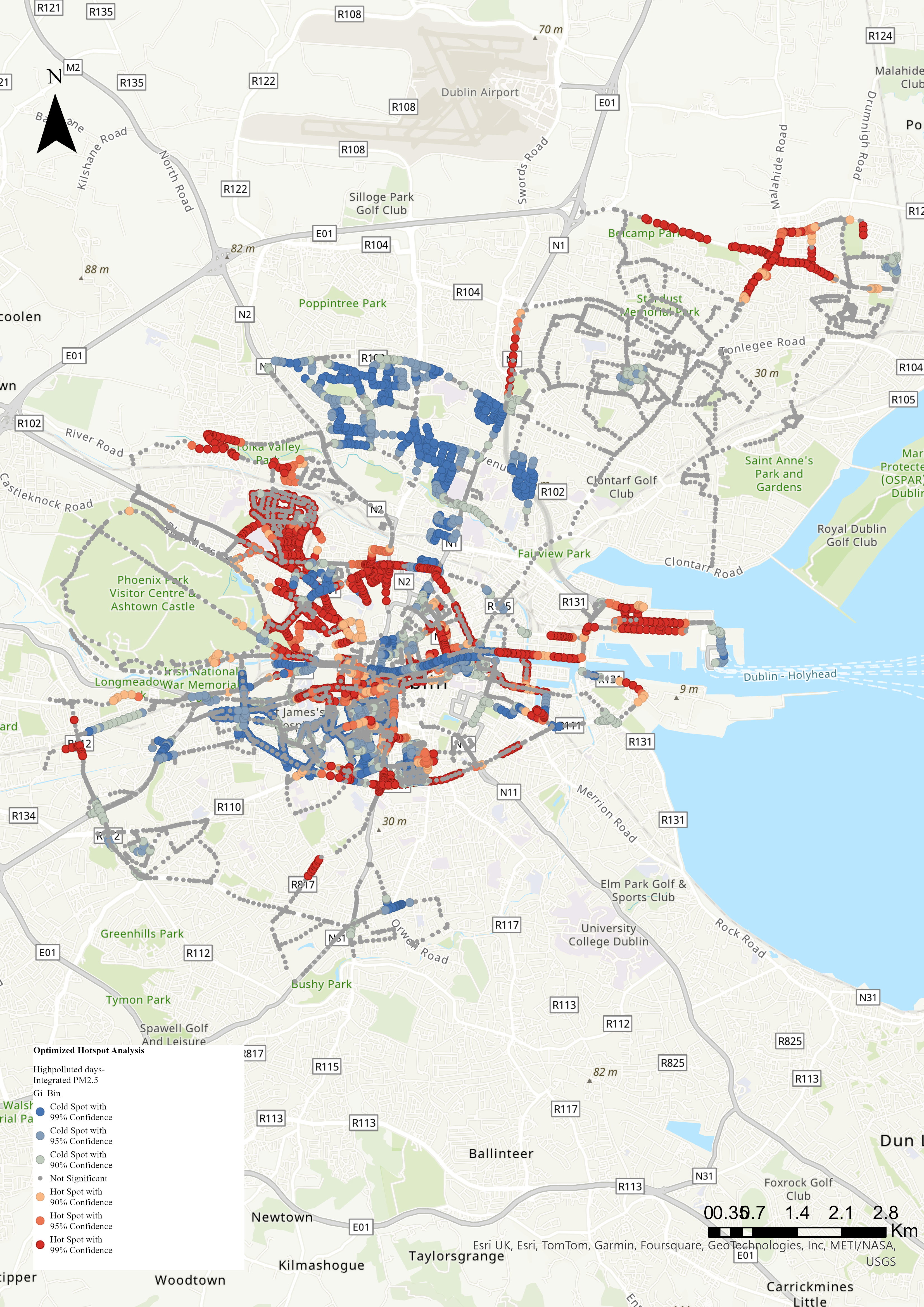

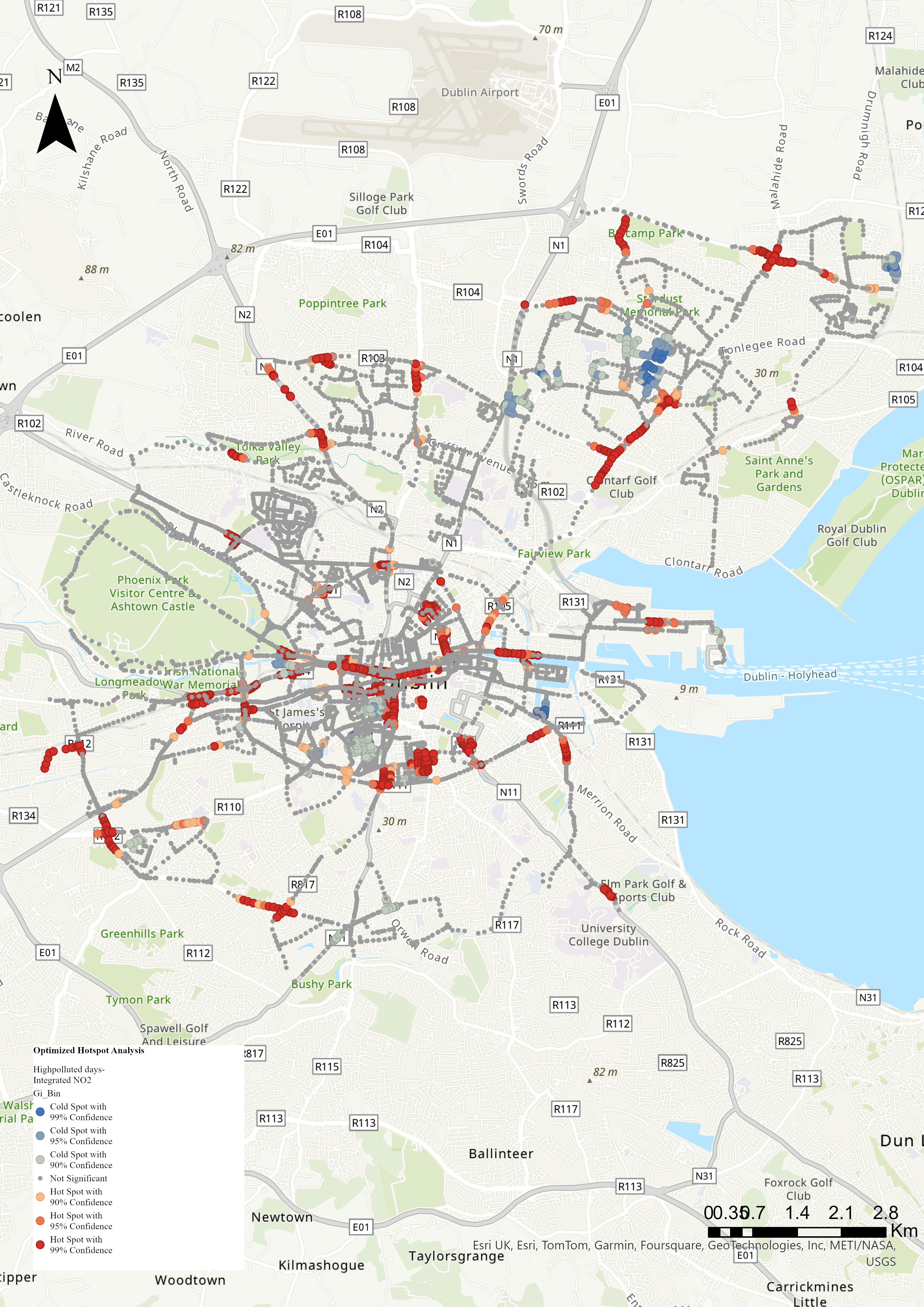


(c)

(b)

**Fig. S8.** Spatial distribution of high pollution levels for (a) PM_2.5_ and NO_2_ together with optimized hotspot analysis for (b) PM_2.5_ and (c) NO_2_ in Dublin. Note: Consolidated information derived from single day outputs were illustrated.

**Table S1**. Instrument performance metrics and limit of detection.

| Pollutant | Bias | Drift | Precision | | | Limit of Detection | | |
| --- | --- | --- | --- | --- | --- | --- | --- | --- |
|  |  |  | 1s | 8s | 170s | 1s | 8s | 170s |
| NO (ppb) | 19% | 22% | 9.7 | 4.0 | 1.0 | 15.6 | 9.6 | 2.2 |
| NO_2_ (ppb) | 2.4 | 4.6 | 4 | 3.1 | 2.1 | 6.4 | 5.2 | 3.4 |
| O_3_ (ppb) | 1.5 | 1.5 | 1.8 | 1.8 | 1.2 | 3 | 2.8 | 1.8 |
| CO (ppm) | 0.03 | 0.03 | 0.028 | 0.026 | 0.023 | 0.028 | 0.02 | 0.012 |
| CO_2_ (ppm) | 5.5 | 18.1 | 3.4 | 3.3 | 3 | 2.4 | 2.2 | 2 |
| PM_2.5_ (μg/m^3^) | 0.6 | 0.6 | 1.4 | 0.9 | 0.5 | 1.6 | 1.0 | 0.4 |

Note: The gas phase instruments were calibrated weekly, and particle monitor was zeroed daily.

**Table S2**. Categories of included ambient monitoring stations in Dublin for comparison.

| Site | Name | Type | Location | Pollutants | Continuous monitoring (Yes/No) | Geolocation |
| --- | --- | --- | --- | --- | --- | --- |
| A | Rathmines | Urban background, National monitoring site | Dublin 6, Dublin | SO_2_, NOx, O_3_, benzene, ozone precursor compounds (VOCs, CO, etc), PM_10_, PM_2.5_ | Yes | 53.3220°N, -6.2672°E |
| B | Dún Laoghaire | Suburban background, National monitoring site | Co. Dublin | NOx, PM_10_, PM_2.5_ | Yes | 53.2860°N, -6.1320°E |
| C | St. John’s Road, Kilmainham | Urban traffic National monitoring site | Kilmainham, Dublin 8, Dublin | NO_x_, PM_10_, PM_2.5_ | Yes | 53.3458°N, -6.2946°E |

**Table S3** Summary statistics of the air pollutants monitored for (a) 1s and (b) 8s rolling median, (c) mean and median concentrations for PM_2.5_ and NO_2_ at ambient monitoring sites, and (d) estimated local pollution for 8s measurement across the study period from Google Airview vehicle.

| **a** | PM_2.5_ (μg/m^3^) | O_3_ (ppb) | NO_2_ (ppb) | NO (ppb) | CO (ppm) | CO_2_ (ppm) |
| --- | --- | --- | --- | --- | --- | --- |
| Minimum | 0.8 | 1.0 | 3.2 | 7.8 | 0.014 | 1.2 |
| 1^st^ | 0.8 | 1.0 | 3.2 | 7.8 | 0.014 | 1.2 |
| 5^th^ | 0.8 | 6.4 | 3.2 | 7.8 | 0.014 | 1.2 |
| 25^th^ | 3.5 | 16.6 | 3.2 | 7.8 | 0.266 | 419.3 |
| Median | 6.0 | 23.5 | 3.2 | 7.8 | 0.307 | 434.6 |
| Mean | 7.8 | 23.0 | 10.3 | 27.4 | 0.335 | 366.8 |
| 75^th^ | 10.0 | 30.4 | 13.4 | 7.8 | 0.369 | 453.0 |
| 95^th^ | 19.8 | 39.6 | 35.6 | 113.2 | 0.590 | 491.0 |
| 99^th^ | 31.9 | 47.7 | 58.6 | 315.8 | 1.022 | 534.1 |
| Maximum | 1960 | 150 | 1827 | 1750 | 21.320 | 2161 |
| N^a^ | 5,030,143 | 1,447,372 | 5,030,143 | 5,030,143 | 5,030,143 | 5,030,143 |

| **b** | PM_2.5_ (μg/m^3^) | O_3_ (ppb) | NO_2_ (ppb) | NO (ppb) | CO (ppb) | CO_2_ (ppm) |
| --- | --- | --- | --- | --- | --- | --- |
| Minimum | 0.5 | 1.4 | 2.6 | 4.8 | 0.089 | 355.0 |
| 1^st^ | 0.8 | 1.4 | 2.6 | 4.8 | 0.217 | 407.3 |
| 5^th^ | 1.6 | 6.3 | 2.6 | 4.8 | 0.238 | 415.9 |
| 25^th^ | 3.7 | 16.4 | 2.6 | 4.8 | 0.274 | 428.4 |
| Median | 6.0 | 23.6 | 6.8 | 4.8 | 0.316 | 440.1 |
| Mean | 7.8 | 23.5 | 12.2 | 25.3 | 0.36 | 446.2 |
| 75^th^ | 9.9 | 30.5 | 17.2 | 15.3 | 0.375 | 457.8 |
| 95^th^ | 19.0 | 39.7 | 38.6 | 114.4 | 0.602 | 495.4 |
| 99^th^ | 30.0 | 47.9 | 63.4 | 301 | 1.044 | 538.9 |
| Maximum | 781 | 141.4 | 1108 | 1727 | 21.0 | 1750 |
| N^b^ | 660,652 | 660,652 | 660,652 | 660,652 | 660,652 | 660,652 |
| > LOD (%) | 99.0 | 97.9 | 44.0 | 69.0 | 100 | 100 |
| % of missing data ^c^ | 38.6 | 27.4 | 23.8 | 37.0 | 38.3 | 25.3 |

Note: ^a^ Data points for the time resolution of 1Hz; ^b^ Number of 8s rolling medians were utilized for calculation; ^c^ Missing data could be due to time when the instrument was not operating properly.

| **c** | St Johns (Urban traffic site) | | Rathmines (urban background) | | Dún Laoghaire (Suburban backgound) | |
| --- | --- | --- | --- | --- | --- | --- |
|  | PM_2.5_ (μg/m^3^) | NO_2_ (μg/m^3^) | PM_2.5_ (μg/m^3^) | NO_2_ (μg/m^3^) | PM_2.5_ (μg/m^3^) | NO_2_ (μg/m^3^) |
| Mean | 7.2 | 41.1 | 5.8 | 16.3 | 6.5 | 18.3 |
| Median | 5.7 | 39.7 | 4.4 | 13.4 | 5.1 | 15.4 |

| **d** | PM_2.5_ Con.(μg/m^3^) | Local contribution to PM_2.5_ (%) | NO_2_ (μg/m^3^) | Local contribution to NO_2_ (%) |
| --- | --- | --- | --- | --- |
| Minimum | 0 | 0 | 0 | 0 |
| 1^st^ | 0 | 0 | 0 | 0 |
| 5^th^ | 0.04 | 2.5% | 0 | 0 |
| 25^th^ | 2.3 | 57.6% | 0 | 0 |
| Median | 4.9 | 74.1% | 12.8 | 47.4% |
| Mean | 6.6 | 66.7% | 58.9 | 42.2% |
| 75^th^ | 9.0 | 84.1% | 50.8 | 78.1% |
| 95^th^ | 17.6 | 91.2% | 266 | 94.9% |
| 99^th^ | 28.3 | 94.3% | 655 | 97.9% |
| Maximum | 4722 | 99.8% | 779 | 99.7% |

**Table S4**. Correlation coefficients for PM_2.5_ and NO_2_ across ambient monitoring sites along with mobile monitoring results over the study period.

| Daytime (i.e. 8 hrs) | NO_2_ (Dun Laoghaire) | NO_2_ (St Johns) | NO_2_ (Rathmines) | NO_2__Daytime | PM_2.5_ (Rathmines) | PM_2.5_ (St Johns) | PM_2.5_ (Dun Laoghaire) | PM_2.5__Daytime |
| --- | --- | --- | --- | --- | --- | --- | --- | --- |
| NO_2_ (Dun Laoghaire) | 1.00 |  |  |  |  |  |  |  |
| NO_2_ (St Johns) | 0.26 | 1.00 |  |  |  |  |  |  |
| NO_2_ (Rathmines) | 0.50 | 0.79 | 1.00 |  |  |  |  |  |
| NO_2__Daytime | 0.24 | 0.49 | 0.50 | 1.00 |  |  |  |  |
| PM_2.5_ (Rathmines) | 0.70 | 0.50 | 0.73 | 0.32 | 1.00 |  |  |  |
| PM_2.5_ (St Johns) | 0.72 | 0.51 | 0.71 | 0.18 | 0.89 | 1.00 |  |  |
| PM_2.5_ (Dun Laoghaire) | 0.77 | 0.51 | 0.69 | 0.23 | 0.87 | 0.88 | 1.00 |  |
| PM_2.5__Daytime | 0.28 | 0.36 | 0.50 | 0.24 | 0.70 | 0.72 | 0.77 | 1.00 |
| 1 hr | NO_2_ (Dun Laoghaire) | NO_2_ (St Johns) | NO_2_ (Rathmines) | NO_2__1 hr | PM_2.5_ (Rathmines) | PM_2.5_ (St Johns) | PM_2.5_ (Dun Laoghaire) | PM_2.5__1 hr |
| NO_2_ (Dun Laoghaire) | 1.00 |  |  |  |  |  |  |  |
| NO_2_ (St Johns) | 0.72 | 1.00 |  |  |  |  |  |  |
| NO_2_ (Rathmines) | 0.60 | 0.68 | 1.00 |  |  |  |  |  |
| NO_2__1 hr | 0.35 | 0.33 | 0.37 | 1.00 |  |  |  |  |
| PM_2.5_ (Rathmines) | 0.07 | 0.21 | 0.46 | 0.12 | 1.00 |  |  |  |
| PM_2.5_ (St Johns) | 0.14 | 0.29 | 0.46 | 0.09 | 0.85 | 1.00 |  |  |
| PM_2.5_ (Dun Laoghaire) | 0.10 | 0.19 | 0.39 | 0.07 | 0.82 | 0.90 | 1.00 |  |
| PM_2.5__1 hr | 0.17 | 0.26 | 0.43 | 0.22 | 0.72 | 0.80 | 0.79 | 1.00 |

Notes: PM_2.5_Daytime_ and NO_2_Daytime_ refer to daytime average measurement from mobile monitoring; PM_2.5__1hr and NO_2__1hr refer to one hour median from mobile monitoring for PM_2.5_ and NO_2_.

**Table S5**. The mean and median mobile monitoring concentrations were calculated using the moving low percentile method for air pollutants across different time windows (8s, 1 hr) and percentiles (1^st^, 5^th^ percentile).

|  | Rolling average/median | PM_2.5_ (μg/m^3^) | NO_2_ (μg/m^3^) | O_3_ (μg/m^3^) | CO  (ppm) | CO_2_  (ppm) |
| --- | --- | --- | --- | --- | --- | --- |
| 1^st^_8 s^a^ | Median ^c^ | 0.8 | 14.2 | 2.8 | 0.246 | 413.4 |
| 5^th^_8 s | Median | 1.7 | 14.2 | 12.5 | 0.273 | 419.7 |
| 5^th^_3 min | Median | 1.8 | 14.2 | 14.9 | 0.277 | 420.4 |
|  | Mean ^d^ | 2.0 | 14.8 | 15.4 | 0.284 | 421.4 |
| 1^st^_1 hr | Median | 1.2 | 14.2 | 2.8 | 0.257 | 415.4 |
|  | Mean | 1.4 | 14.5 | 6.2 | 0.272 | 416.4 |
| 5^th^_1 hr | Median | 1.8 | 14.2 | 16.5 | 0.284 | 422.1 |
|  | Mean | 2.4 | 25.4 | 16.8 | 0.308 | 424.1 |
|  | Median, mean (SD ^b^) | 1.6, 1.5(0.5) | 15.4, 14.2(3.5) | 9.4, 9.6(6.2) | 0.273, 0.271(0.019) | 418.1, 418.1(3.9) |
| 1 min_Minimum | Mean | 6.7 | 29.7 | - | - | - |
| 1 hr_Minimum | Mean | 4.9 | 16.2 | - | - | - |
| Daytime_Minimum | Mean | 5.8 | 19.2 | - | - | - |

*Notes*: ^a^8 s: median concentration grouped by 50 m road segments.

^b^SD refers to standard deviation derived from different moving averages and median schemes.

^c^ Median refers to the rolling median used for calculation.

^d^ Mean refers to the rolling averages that were used for calculation.
